# Ribose operon repressor (RbsR) contributes to the adhesion of *Aeromonas hydrophila* to *Anguilla japonica* mucus

**DOI:** 10.1002/mbo3.451

**Published:** 2017-01-27

**Authors:** Guifang Lin, Wenbo Chen, Yongquan Su, Yingxue Qin, Lixing Huang, Qingpi Yan

**Affiliations:** ^1^ State Key Laboratory of Large Yellow Croaker Breeding Ningde Fujian China; ^2^ Key Laboratory of Healthy Mariculture for the East China Sea Fisheries College Ministry of Agriculture Jimei University Xiamen Fujian China; ^3^ College of Ocean & Earth Sciences Xiamen University Xiamen Fujian China

**Keywords:** *A. hydrophila*, adhesion, intracellular survival, Mini‐Tn*10*Km, *rbsR*

## Abstract

The characterization of adhesion between pathogenic bacteria and the host is critical. Pathogenic *Aeromonas hydrophila* was shown to adhere in vitro to the mucus of *Anguilla japonica*. To further investigate the adhesion mechanisms of *A. hydrophila*, a mini‐Tn*10* transposon mutagenesis system was used to generate an insertion mutant library by cell conjugation. Seven mutants that were impaired in adhesion to mucus were selected out of 332 individual colonies, and mutant M196 was the most severely impaired strain. National Center for Biotechnology Information (NCBI) blast analysis showed that mutant M196 was inserted by mini‐Tn*10* with an ORF of approximately 1,005 bp (GenBank accession numbers KP280172). This ORF is predicted to encode a protein consist of 334 amino acid, which displays the highest identity (98%) with the RbsR of *A. hydrophila* ATCC 7966. Random inactivation of *rbsR* gene affected the pleiotropic phenotypes of *A. hydrophila*. The adhesion ability and the survival level of the *rbsR* gene mutant (M196) were attenuated compared with the wild‐type and *rbsR* complementary type. The findings of this study indicated that RbsR plays roles in the bacterial adhesion and intracellular survival of *A. hydrophila*.

## 1 Introduction

Adhesion to the host external surfaces and tissues by pathogenic bacteria represents the first crucial step in most infections, and it had been regarded as an important virulence factor in colonizing the host surface for proliferation and for the release of virulence factors of many pathogenic bacteria (Grześkowiak, Collado, Vesterlund, Mazurkiewicz, & Salminen, 2011; Huang et al., 2016; Wang et al., 2015).The ability of pathogens to attach to the host mucus, cells, and other tissues is mediated by adhesions that can cause many infectious diseases or other harm (Hori & Matsumoto, 2010). Therefore, the inhibition of bacterial adhesion to the host could be useful therapeutically (Acord, Maskell, & Sefton, 2005). It is important to understand the mechanisms of bacterial adhesion as well as the major factors controlling this attachment. Recently, the adhesion of many clinical pathogens to their hosts has been elucidated, and bacterial adhesion was shown to be mostly influenced by motility, cell hydrophobicity, and bacterial surface structures (Chen, Yan, Wang, Zhuang, & Wang, 2008; He et al., 2015; Losensky, Vidakovic, Klingl, Pfeifer, & Fröls, 2015). Experiments with the pathogens *Pseudomonas aeruginosa*,* Vibrio alginolyticus; Vibrio vulnificus,* and *Legionella pneumophila* (Huang, Hu et al., 2015; Kim & Rhee, 2003; Luo et al., 2016; Olga, Jens, & Michael, 2011; Shao et al., 2013) demonstrated the important role of flagella or lipopoly saccharides in establishing the initial interaction with mucosal surfaces or cells and that this defect can reduce the ability of adhesion. In addition, the genetic basis of colonization has been studied in *V. vulnificus*,* L. pneumophila,* and *Streptococcus suis* (Chang et al., 2005; Kim & Rhee, 2003; Zhang et al., 2015) and in the genes that impact bacterial adhesion that can also contribute to bacterial pathogenesis. In the pathogenic bacteria *V. vulnificus*, FlgC was shown to be involved in adhesion to HeLa cells, and the *flgC* mutation could decrease the level of adhesion, cytotoxicity, and lethality in mice (Kim & Rhee, 2003). Intracellular bacteria *L. pneumophilalaia* was shown to be involved in adhesion to the human lung alveolar epithelial cell line A549, and the *laiA* gene mutation could cause reduced mortality in A/J mice (Chang et al., 2005). The *dnaJ* of *S. suis* Type 2 was important for adhesion to host cells, and the *dnaJ*PKO strain can result in reduced adhesion to cultured cell monolayers (Zhang et al., 2015). These pathogenic genes were all considered to be the key factors for bacterial adhesion and pathogenicity.

*Aeromonas hydrophila* has been recognized as a gram‐negative bacillus of the Aeromonadaceae family, and it can survive in a wide variety of aquatic systems (Cao et al., 2012). It is an opportunistic pathogen and can infect a great variety of poikilothermic and homeothermic animals, including humans (Krovacek et al., 1994; Mccoy et al., 2010; Sime‐Ngando, 2015). Several structures have previously been demonstrated to be related to the pathogenicity of *A. hydrophila*, including adhesion (Grześkowiak et al., 2011; Hori & Matsumoto, 2010; Van Der Marel, Schroers, Neuhaus, & Steinhagen, 2008). *A. hydrophila* attached strongly to the host surface using flagella‐promoted motility in the optimum environment, and the environmental factors obviously affected bacterial adhesion (Benhamed, Guardiola, Mars, & Esteban, 2014). Several genes essential for *A. hydrophila* adhesion have been identified. The *A. hydrophila flgE* gene mutation can cause the absence of flagella, and the mutant bacteria can exhibit inadequate motility, adhesion, invasion, and survival in host macrophages when compared with the wild‐type B11 and the complementary strain (Qin et al., 2014). Single gene mutations of *minD* can lead to flagella deficiency and adhesion reduction (Huang, Qin et al., 2015).

It has been extensively reported that some genes associated with flagella play an important role in *A. hydrophila* adhesion. However, few studies have focused on fully exploring the adhesion‐related genes of the pathogenic bacteria *A. hydrophila*, especially genes related to nutrient metabolism or enzymes. The aim of this study was to identify more adhesion‐related genes of pathogenic *A. hydrophila* with random gene inactivation method and to probe other pleiotropic phenotypes of *A. hydrophila* by constructing mutant and complementary strains to study the mechanisms of these genes.

## 2 Experimental procedures

### 2.1 Bacterial strains and growth conditions

The bacterial strains and plasmids used in this study are listed in Table 1. Pathogenic *A. hydrophila* was isolated from the naturally infected *Anguilla japonica*, Matrix‐Assisted Laser Desorption/ Ionization Time of Flight Mass Spectrometry (MALDI‐TOF‐MS) and sequencing of 16SrRNA, and confirmed as a pathogen by artificial infection. *A. hydrophila* and *Escherichia coli* were grown in Luria‐Bertani Miller broth (LB) at 28°C and 37°C, respectively. The bacterial sample was centrifuged at 4,000 g at 4°C for 5 min, and bacterial cells were resuspended in phosphate‐buffered saline (PBS, pH 7.4) after incubation for 18 hr. The medium was supplemented with the appropriate antibiotics at the following concentrations: 600 μg/ml kanamycin (Km), 100 μg/ml ampicillin (Amp), 100 μg/ml streptomycin (Sm), and 34 μg/ml chloramphenicol (Cm).

**Table 1:** Strains and plasmids used in this study

Strain or plasmid	Characteristic(s)	Source or reference
Strains		
*A. hydrophila*		
W1	wild‐type strain	
M01 ~ M332	mini‐Tn*10*Km insertion mutant (SmR KmR)	This study
M196	*rbsR*: mini‐Tn*10*Km (SmR KmR)	This study
MC196	WM196 complemented with pACYC184‐*rbsR* (CmR)	This study
*Escherichia coli*		
SM10	*thi thr leu tonA lacY supE recA* RP4‐2‐Tc::Mu::Km (λpir)F‐, 80dlacZ ΔM15, Δ(*lacZYA‐argF*) U169, *deoR, recA1*	(Herrero et al., 1990)
*E. coli* DH5α	*endA1, hsdR17* (rK−, mK+), *phoA*, supE44, λ‐, *thi‐1*,* gyrA96*,* relA1*	
Plasmid		
pMD18‐T	Cloning vector (ApR)	Takara
pLOF/Km	Tn*l0*‐based delivery plasmid with (KmR ApR);	(Herrero et al., 1990)
pACYC184	(CmR TcR)	provided by Prof. Nie
pACYC184‐rbsR	pACYC184 derivative containing 1,005 bp fragment of rbsR putative promoter and ORF(CmR)	This study

### 2.2 Preparation of *A. japonica* mucus and *A. hydrophila* adhesion in mucus in vitro

A group of five healthy *A. japonica* were purchased from an aquatic product market in Xiamen of Fujian Province. The skin mucus was prepared using a modification of a previously described technique (Huang, Qin et al., 2015). Briefly, the skin mucus gel was collected by scraping the skin surfaces with a rubber spatula, and the gel was homogenized in PBS and centrifuged twice at 20,000 g at 4°C for 30 min to remove particulates. The supernatant was filtered sequentially through .45 and .2 μm pore size filters. The resulting mucus sample was adjusted to 1 mg of protein/ml with sterile aged seawater, and the protein concentration was determined using the method that was described by Bradford (Bradford, 1976).

Gill mucus was prepared using a technology we described before (Chen et al., 2008). Blood was completely removed from the caudal vessel, and the gill arches were excised and soaked in PBS for 2 hr at 4°C, with occasional shaking. The mucus preparation was centrifuged twice at 20,000 g for 30 min at 4°C to remove particles and cellular material, followed by filtration of the final supernatant through .45 and .2 μm pore size filters. The protein concentration of mucus preparation was adjusted to 1 mg/ml in PBS. The protein concentration was determined using the method of Bradford (1976).

Intestinal mucus was prepared using a technique we described before (Yan, Chen, Ma, Zhuang, & Wang, 2007). The fish were starved for 48 hr without feeding and the intestines were then removed and divided into foregut and hindgut according to intestinal morphology. The guts were transferred to sterile petri dishes and washed by sterile .01 mol/L PBS (pH 7.2). Next, they were split open with a scalpel, and the foregut and hindgut mucus were collected by scrapping of the inner surface of the intestines with a rubber spatula to remove the layer of mucus gel covering the intestinal lumen, respectively. The mucus was homogenized in .01 mol/L PBS (pH 7.2).The mucous preparations were centrifuged twice at 20,000 g for 30 min at 4°C to remove particulate and cellular material, followed by filtration of the final supernatant through .45‐ and .2‐μm pore size filters. The mucus samples were adjusted to 1 mg protein/ml with PBS. The protein concentration was determined using the method of Bradford (1976).

An in vitro adhesion assay was performed using an indirect enzyme‐linked immunosorbent assay (ELISA) method with 96‐well microtiter polystyrene plates, as previously described and modified (Qin et al., 2014). Briefly, 100 μl of the mucus samples were added to each well, the mucus was fixed by incubation overnight at 4°C, and unbound mucus was removed by washing the wells twice with 200 μl of PBS. Equal volume aliquots of bacteria were added to the wells and incubated at 28°C for 2.5 hr. Then, adhered bacterial cells to mucus were removed by washing the wells twice with 200 μl of PBS, and the bacteria were dried at 60°C. The wells were washed three times with 200 μl of phosphate‐buffered saline Tween‐20 (PBST), and all wells were blocked with fetal bovine serum (1:10). Subsequently, a sample (100 μl) of the indicated dilution of rabbit antibacterial serum in PBS was added to each well after washing the wells three times with 280 μl of PBST, incubating for 1 hr at 37°C, and washing three times with PBST. Horseradish peroxidase (HRP) goat anti‐rabbit immunoglobulin G diluted (1:1,000) in PBS was then added to each well and incubated for 1 hr at 37°C. After washing four times with PBST, 100 μl of a fresh OPD‐H_2_O_2_ was added to each well. The color was allowed to develop for 30 min and was then measured at 492 nm with a microtiter plate reader (Bio‐Tek). Controls consisted of wells without bacteria to ensure that the primary antibody did not cross‐react with the endogenous bacteria or with the host cells and wells without the immobilized cells to determine the amount of nonspecific binding of the bacteria to the plastic.

To further confirm the result of bacterial adhesion assay, a total of 50 μl of mucus was evenly spread on a 22 mm × 22 mm glass slide area and fixed with methanol for 20 min. Then, 1 ml of bacterial suspension (10^8^ CFU/ml) was placed on the mucus‐coated glass slides, incubated for 2 hr at 25°C in a humidified chamber, and washed with PBS five times. Lastly, the bacteria were fixed using 4% methanol for 30 min, dyed via crystal violet for 3 min, and counted under a microscope (×1,000). Each group was conducted in three trials, and 20 fields of view were selected. Negative control was performed using PBS instead of bacterial suspension.

### 2.3 Mini‐Tn10 mutagenesis of *A. hydrophila* strain W1 and genetic screening for adhesion‐deficient mutants

The mini‐Tn10 Km transposon was transformed into *A. hydrophila* wild strain W1 on the suicide vector pLOF/Km carried by *E. coli* Sm10 through a filter mating technique that had been previously developed (Qin et al., 2014). Briefly, the suicide vector mini‐Tn10 Km (KmR and AmpR) was extracted from *E. coli* CC118 and transferred into *E. coli* Sm10. Then, the *A. hydrophila* wild‐type (W1) and *E. coli* Sm10 pLOF/Km (KmR and AmpR) were combined with Isopropyl β‐D‐1‐thiogalactopyranoside (IPTG) induction. Serial dilutions of the mating mixture were plated on LB supplemented with kanamycin to select the mutants. The mutants in the genes were identified by gene walking polymerase chain reaction (PCR, Takara).

### 2.4 Southern blotting

A single insertion event in the rbsR::Km mutant was confirmed. A single chromosomal transposon insertion in the rbsR::Km mutant was confirmed with genomic DNA prepared from the rbsR::Km mutant using the standard method (Rock & Nelson, 2006). Briefly, DNA was extracted from the bacterial cells by proteinase K‐sodium dodecyl sulfate digestion followed by phenol‐chloroform purification. Approximately 4 g of genomic DNA of wild type and rbsR::Km mutant were digested with *Sal* I. A total of 10 μg of DNA was digested to completion with *Sal* I and run on agarose gels. Southern blots were prepared on Hybond nylon filters. Oligonucleotide probes end‐labeled with a Dig‐High Prime DNA Labeling and Detection Starter Kit II were used for detection according to the manufacturer's protocol. A 176‐bp STM PCR product was amplified using primers Tn10kan (CGGGGATCGCAGTGG) and Tn10kan (TGGGAAGCCCGATGC) and labeled using a Dig‐High Prime DNA Labeling and Detection Starter Kit II. The probe was hybridized to the genomic DNA on the membrane and detected using the Dig‐Highreagents in accordance with the manufacturer's instructions.

### 2.5 Sequence analysis of the mini‐Tn*10* insertion mutants

Thermal asymmetric interlaced polymerase chain reaction (TAIL‐PCR) (Singer & Burke, 2003) was used to obtain the mutant DNA sequences flanking the transposon to find the insertion sites in *A. hydrophila*. The arbitrary primers were provided by a genomic walking kit (Takara, Bio). The specific transposon nested primers are listed in Table 2. LSP4, LSP5, and LSP6 were used to amplify upstream sequences flanking the transposon, and RSP4, RSP5, and RSP6 were used to amplify downstream sequences flanking the transposon. TAIL‐PCR products were purified with a Gel Extraction Kit (Omega, Bio‐Tek), cloned into a pMD18‐T vector (Takara, Bio) and sequenced. DNA sequences were analyzed by BLASTN (National Center for Biotechnology Information, Bethesda, MD, USA) and other software such as DNA Star.

**Table 2:** The specific primers used in TAIL‐PCR

Primer	Sequence
LSP4	5′ ATGCTTGATGGTCGGAAGAGGC 3′
LSP5	5′ CATCGGGCTTCCCATACAATCG 3′
LSP6	5′ ATTATCGCGAGCCCATTTATACCC 3′
RSP4	5′ CCTGTTGAACAAGTCTGGAAAGAAATG 3′
RSP5	5′ GATCTTGCCATCCTATGGAACTG 3′
RSP6	5′ TTACGCTGACTTGACGGGACGG 3′

### 2.6 Plasmid complementation

For complementation studies, *A. hydrophila* wild‐type DNA fragments with *rbsR* were PCR amplified using primers *rbsR*‐EcoRV‐For (5′‐GAT ATC CTA ACC AGA GAG GGT GGA ATA GTG C‐3′) and *rbsR*‐2HA‐SphI–Rev (5′‐GCA TGC *TTA* *CTA GAG GCT AGC ATA ATC AGG AAC ATC ATA CGG ATA* GCG CAG TGA CTT GAC CGA GC‐3′) (enzyme restriction sites are underlined, the HA‐tag sequence is in italic and the stop codon is underlined and in italic). Amplified fragments of 1,005‐bp were ligated into pMD18‐T (Takara). The DNA insert was recovered by *BamH*I and *Sph*I restriction digestion and was ligated into *BamH*I and *Sph*I phosphatase‐treated pACYC184 to generate plasmid pACYC‐*rbsR*. This vector was transferred into *E. coli* DH5α, and recombinant plasmids surviving on chloramphenicol plates were selected by tetracycline sensitivity. Then, the recombinant plasmids were electroporated into a *rbsR* mutant strain, and bacteria complementary strains surviving on chloramphenicol plates were selected from tetracycline sensitivity.

Whole‐cell proteins were obtained from Aeromonas strains grown at 28°C. Equivalent numbers of cells were harvested by centrifugation, and each cell pellet was suspended in 50–200 μl of sodium dodecyl sulfate‐polyacrylamide gel electrophoresis loading buffer and boiled for 10 min. The expression of *rbsR* was detected by western blot according to the supplier's instructions with an anti‐HA tag antibody that was appropriate for the method (Green & Sambrook, 2012). Following sodium dodecyl sulfate‐polyacrylamide gel electrophoresis and transfer to nitrocellulose membranes, the membranes were blocked with skimmed milk powder (5 mg/ml) and probed with hemagglutinin (HA)‐tag antibodies (1:1,000). The unbound antibody was removed by three washes in PBST, and a goat anti‐rabbit HRP‐conjugated secondary antibody (1:100) was added. The unbound secondary antibody was removed by three washes in PBST. The bound conjugate was then measured using a Super Signal West Pico Trial kit (Thermo, USA) and electrochemiluminescence (ECL) western blot system (LAS‐4000 mini, Fuji, Japan) according to the manufacturer's instructions.

### 2.7 Preparation of macrophages, and in vitro invasion and survival

Tilapia macrophages were prepared, and in vitro invasion and survival of *A. hydrophila* in tilapia macrophages were conducted. Healthy tilapia (*Oreochromis niloticus*) individuals were obtained from an aquaculture farm. *O. niloticus* macrophages were prepared as Qin et al. (Qin et al., 2014) described with some modification. Briefly, *O. niloticus* was bled by syringe as completely as possible from the caudal circulation to reduce the blood volume in the pronephros, and then the surface of the fish was sterilized with 70% alcohol. The tissue overlaying the pronephros was removed and then placed in 2 ml of ice‐cold L‐15 medium (HyClone Laboratories, Inc., Utah, USA) supplemented with 100 IU S/P ml^−1^ and 2% fetal bovine serum. The tissues of pronephros were sheared and pushed through a 100 μm nylon mesh. The cell suspension was layered onto a 34%/51% discontinuous Percoll (GE Healthcare Bio‐Sciences AB, Sweden) density gradient with a syringe and centrifuged at 400 × g for 30 min at 4°C. The band of cells lying at the interface of the 34%/51% Percoll gradient was collected, washed twice and the living and dead cells were counted by trypan blue staining. Then, the cells were counted with hemocytometers and adjusted to 2.4 × 10^7^ cells ml^−1^ in a L‐15 medium with 5% FBS, 100 IU S/P ml^−1^ and 10 IU heparin ml^−1^ and transferred to 6‐well plates, 1 ml/well.

In vitro bacterial invasion tests were performed as previously described (Martin & Mohr, 2000) with some modification. Briefly, this assay was performed according to the following steps. First, 1 ml of the macrophage suspension was added to each well of 6‐well culture plates and incubated for 0.5 hr. Then, an equal volume of bacterial suspension with a multiplicity of infection of 100 (100 bacteria per macrophage added) was added to each well and incubated at 28°C. After 1 hr of bacterial invasion, the cell suspensions were treated with 2,500 μg of gentamicin ml^−1^ for 20 min at 28°C to kill the extracellular bacteria, and the supernatant was carefully removed without disturbing the packed cells. Then, fresh L‐15 medium with 10 IU heparin ml^−1^, 10% FBS and 100 units S/P was added to the packed cells and allowed to incubate at 28°C in 5% CO_2_ with the time point denoted as 0 hr. After incubation for 1 hr, the cells were centrifuged for 5 min at 400 ***g*** at 28°C, and the supernatant was aspirated. Then 1 ml of sterile distilled water was added for 30 min to lyse the cells. The CFU number of the cell lysate was determined by plate counting.

### 2.8 Statistical analyses

All data were statistically analyzed with SPSS16.0 (SPSS, Chicago, IL, USA). The mean ± standard deviation was calculated for each sample. Unless otherwise stated, all experiments were performed at least three times in triplicate assays.

## 3 Results

### 3.1 Adhesion‐reduced mutant isolation

**Figure 1:**
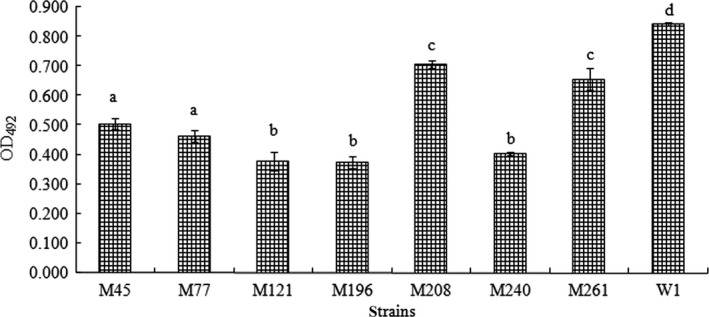
Adhesion ability of wild‐type (W1) and seven mutant strains of *A. hydrophila* to fish mucus. The results are expressed as the optical density (OD) recorded at 492 nm in a microplate reader (mean and *SD*). Values denoted by different letters were significantly different when compared by analyses of variance (P < 0.05)

A mini‐Tn10 transposon mutagenesis system was used to generate an insertion mutant library by cell conjugation. A mutagenesis library with 332 random insertion mutants of *A. hydrophila* was constructed, and each mutant in the library was subjected to the adhesion assay. Seven mutants were found to be reduced in adhesion to *A. japonica* skin mucus compared with W1, and the mutant strain (M196) was the most significantly inadequate regarding adhesion to *A. japonica* mucus (Figure 1).

### 3.2 Southern blotting

**Figure 2:**
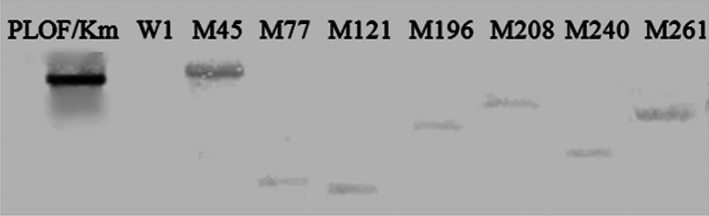
Detection of a mini‐Tn*10* insertion in the wild‐type strain (W1) and the mutants by Southern blotting; a single band was present in all mutants and in the plasmid positive control pLOF/Km

Southern blotting analysis showed that a single band was present in all mutants and the plasmid positive control pLOF/Km. No signal was detected in the negative control wild‐type strain W1 (Figure 2), which confirmed that the mutations were caused by the insertion of transposon mini‐Tn*10*Km and that only a single transposon insertion was present in the chromosome for each mutant.

### 3.3 Mini‐Tn*10* insertion site in the mutant strain (M196)

**Figure 3:**
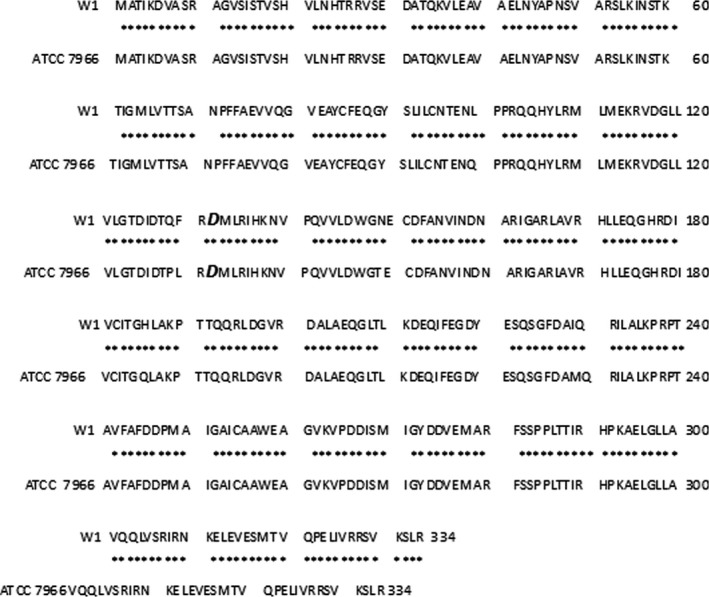
Blast rbsR of *A. hydrophila (W1)* and *A. hydrophila subsp. hydrophila* ATCC7966. The Tn10 insertion site is indicated with a bold, enlarged letter

Sequence analysis showed that the mini‐Tn*10* insertion site was a 1,005 bp ORF that shared the highest identity (98%) with the *rbsR* gene of *A. hydrophila* ATCC 7966 (accession no: CP000462.1). This *rbsR* gene encoded a ribose operon, which is a repressor protein that was predicted to contain 334 amino acid residues with a predicted 33 kDa molecular weight. The Tn*10* insertion site is indicated with a bold and enlarged letter (Figure 3).

### 3.4 RbsR protein expression in the complementary strain

**Figure 4:**
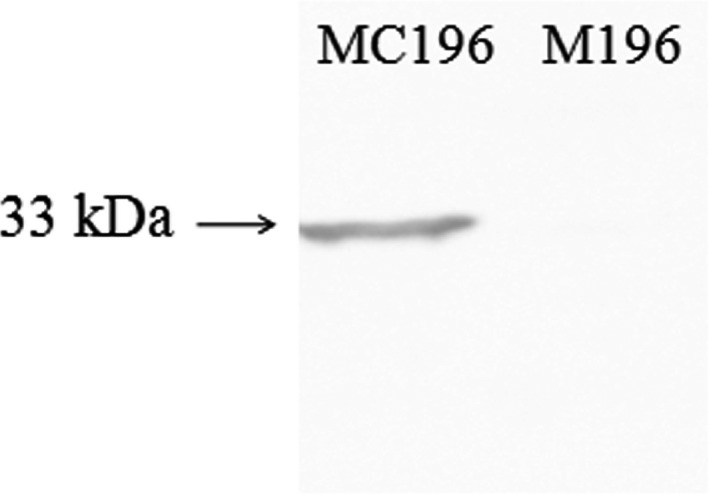
Detection of RbsR protein expression in the complementary strains to screen for positive complementary strains (MC196). Lane 1 shows that *rbsR*‐HA was expressed at approximately 33 kDa in the complementary strain MC196, so the strain of lane 1 was identified as the complementary strain that was needed. Lane 2 shows that the mutant strain M196 had no single line, which was a negative control

After the recombinant expression plasmid pACYC184‐*rbsR* was introduced into the mutant (M196), the expression of protein RbsR in the complementary strain (MC196) was detected by western blotting. Western blotting analysis showed that a positive signal of approximately 33 kDa was detected, which confirmed that the fusion protein *RbsR*‐HA was expressed in the complementary strain (MC196) but not in the mutant strain (M196; Figure 4).

### 3.5 Adhesion assay

**Figure 5:**
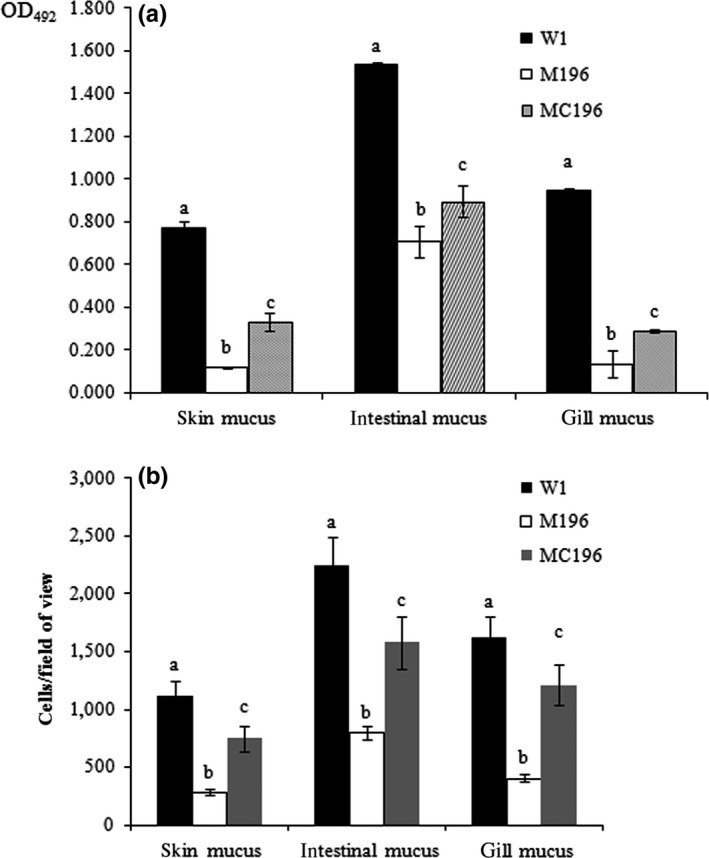
Bacterial adhesion assay with ELISA (a) and microscopy (b). Values denoted by different letters were significantly different when compared to W1. The adhesion ability to skin, intestinal and gill mucus of the mutant strains were significantly inadequate when compared with the wild‐type (W1) and complementary strains (MC196; *P *<* *.05). Values denoted by different letters were significantly different when compared with an analyses of variance (*P *<* *.05)

Adhesion of the wild‐type (W1), the *rbsR* mutant strain (M196), and the complementary strain (MC196) to the host skin, intestinal and gill mucus was detected. The results showed that the adhesion of the M196 bacteria to skin, intestinal and gill mucus was approximately 15%, 46% and 14%, respectively, compared with that of the wild‐type (W1) strain. The adhesion of bacteria to the skin, intestinal and gill mucus with the complementary strain recovered to more than 43%, 59%, and 35% of that of the wild‐type strain, respectively (Figure 5). These data suggested that the mutation in *rbsR* significantly affected bacterial adhesion.

### 3.6 Intracellular survival in macrophages

**Figure 6:**
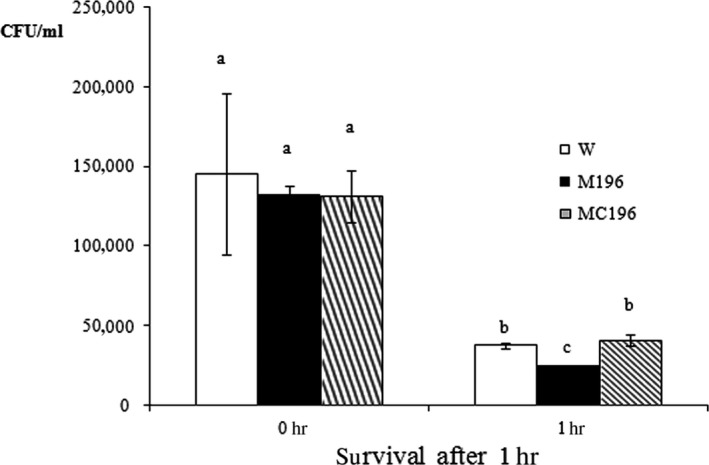
Bacterial invasion and survival level in fish macrophages. The survival number of the mutant strain (M196) was significantly lower than that of the wild‐type (W1) and the complementary strain (MC196) after 1 h. Values denoted by different letters were significantly different when compared with an analyses of variance (*P *<* *.05)

Given the importance of *rbsR* in *A. hydrophila* virulence, the number of invasive and surviving bacteria of the wild‐type (W1), *rbsR* mutant (M196), and complementary strains (MC196) in macrophages was determined in vitro. The results at 0 hr show the number of invasive bacteria into macrophages, and the results at 1 hr represent the number of surviving bacteria after entry into the macrophages for 1 hr. The data showed that there was no difference between the number of invasive wild‐type bacteria, mutant strain and that of the complementary strain, while the survival number of the mutant strain (M196) was significantly lower than that of the wild‐type (W1) and the complementary strain (MC196) after 1 hr (Figure 6). Furthermore, there was only approximately 17% of invasive mutant bacteria that survived in macrophages 1 hr after entry into the cells, while the survival rates of wild‐type (W1) and the complementary strain (MC196) were ~26% and ~31%, respectively, which suggested that the survival of the mutant bacteria in host macrophages were seriously impaired.

## 4 Discussion

Recently, adhesion has been found to be associated with the virulence of many types of pathogens (Benhamed et al., 2014; Luo et al., 2016). Some studies have suggested that bacterial adhesion genes, such as *flrA*,* flrB*, and *flrC* can mostly be related to flagella. Bacterial adhesion is an extremely complicated process and can be controlled by one or more genes. To understand the genetic basis of adhesion in the *A. hydrophila* prototype, transposon insertion mutants that were inadequate in adhesion isolated were characterized. In this study, pathogenic *A. hydrophila* W1 could adhere to *A. japonica* mucus well, and a gene corresponding to a repressor protein was identified: *rbsR* (M196).

Previous studies have found a few genes, such as *CtBP*, which is not traditionally considered as an adhesion factor, that can impact adhesion (Grooteclaes & Frisch, 2000). The results confirmed the screening data in this study.

In this study, the mutant strain (M196) was significantly inadequate regarding adhesion to *A. japonica* mucus and the complementary strain (MC196) recovered the adhesion ability to some extent. rbsR deletion can immediately affect the mRNA level of the whole rbsRACBD (rbs) operon, and the gene products of the rbs operon of *Corynebacterium glutamicum* encode a ribose‐specific ATP‐binding cassette (ABC) transport system with corresponding regulatory proteins (i.e., rbsR). ABC transporters are membrane proteins that couple the energy from ATP hydrolysis to transport substrates, including nutrients and toxins (Chen, Lu, Lin, Davidson, & Quiocho, 2003; Nentwich et al., 2009). Previous reports have shown that NCAM‐mediated adhesions may be modulated by extracellular ATP (Skladchikova, Ronn, Berezin, & Bock, 1999). Potassium requires ATP to stimulate the adhesion of *P. aeruginosa*; ATP synthesis may play an important role in the adhesion process (Espinosa‐Urgel, Salido, & Ramos, 2000). Mostly, nutrients influence the expression of adhesion and invasion proteins in *Listeria monocytogenes*, and a starvation assay that inhibited *V. alginolyticus* adhesion to the fish mucus showed that the nutrients were important to bacteria adhesion (Chen et al., 2008; Jaradat & Bhunia, 2002). It can be deduced that RbsR may affect the expression of ABC transporters that couple the energy from ATP hydrolysis to transport nutrients and that the nutrients can affect the bacterial adhesion. Although specific mechanisms on how RbsR functions in ATP and some adhesion processes remain uncertain, our findings provide an important new understanding of this process.

Once bacteria have irreversibly attached to a surface, the process of biofilm maturation begins (Dunne, 2002). Cells within the biofilm can modulate cytokine synthesis and can remain hidden from antibodies and complement factor recognition and from subsequent host cell phagocytosis (Cortés, Consuegra, Sinisterra, & Mendez‐Vilas, 2011; McCann, Gilmore, & Gorman, 2008). *Brucellasuis* releases compounds, such as adenine or GMP, that can inhibit phagocyte functions and contribute to the intracellular survival of the bacteria (Foulongne, Bourg, Cazevieille, Michaux‐Charachon, & O'Callaghan, 2000). RbsR protein has been believed to be an ordinary LacI‐type repressor for a single target rbsDACBK operon, which may be involved in the production of GMP in nucleotide synthesis (Shimada, Kori, & Ishihama, 2013). Mostly, the *E. coli rbsR* gene was found to be a transcriptional repressor for the ribose operon that is involved in the regulation of purine nucleotide synthesis and nutrient synthesis and transport (Daigle, Graham, & Curtiss, 2001; Shimada et al., 2013). In this study, the data suggested that the survival ability of the *rbsR* mutant in host macrophages was severely impaired. This impairment could be related to the result that *Salmonella typhi rbsR* mutations could decrease the level of survival within human macrophages at both 2 and 24 hr after bacterial infection (Ocallaghan, Maskell, Liew, Easmon, & Dougan, 1988). We hypothesized that the *A. hydrophila rbsR* mutation could affect nutrient synthesis and transportation causing poor survival ability. However, further research is still necessary. Our future research efforts will focus on the strategies that permit *A. hydrophila* intracellular survival within phagocytes.

In summary, our study showed that *rbsR* mutations affected *A. hydrophila* behavior related to virulence, such as adhesion and intracellular survival ability.

## Conflict of interest

The authors declare no potential conflicts of interest.

## References

[mbo3451-bib-0001] Acord, J. , Maskell, J. , & Sefton, A. (2005). A rapid microplate method for quantifying inhibition of bacterial adhesion to eukaryotic cells. Journal of Microbiological Methods, 60, 55–62. 15567225 10.1016/j.mimet.2004.08.011

[mbo3451-bib-0002] Benhamed, S. , Guardiola, F. A. , Mars, M. , & Esteban, M. A. (2014). Pathogen bacteria adhesion to skin mucus of fishes. Veterinary Microbiology, 171, 1–12. 24709124 10.1016/j.vetmic.2014.03.008

[mbo3451-bib-0003] Bradford, M. M. (1976). A rapid and sensitive method for the quantitation of microgram quantities of protein utilizing the principle of protein‐dye binding. Analytical Biochemistry, 72, 248–254. 942051 10.1016/0003-2697(76)90527-3

[mbo3451-bib-0004] Cao, Y. A. , He, S. X. , Zhou, Z. G. , Zhang, M. C. , Mao, W. , Zhang, H. T. , … Yao, B . (2012). Orally administered thermostable n‐acyl homoserine lactonase from bacillus sp strain AI96 attenuates *Aeromonas hydrophila* infection in zebrafish. Applied and Environmental Microbiology, 78, 1899–1908. 22247159 10.1128/AEM.06139-11 PMC3298178

[mbo3451-bib-0500] Chang, B. , Kura, F. , Amemura‐Maekawa, J. , Koizumi, N. , & Watanabe, H . (2005). Identification of a novel adhesion molecule involved in the virulence of Legionella pneumophila. Infection and immunity, 73, 4272‐4280. 15972519 10.1128/IAI.73.7.4272-4280.2005 PMC1168565

[mbo3451-bib-0005] Chen, J. , Lu, G. , Lin, J. , Davidson, A. L. , & Quiocho, F. A. (2003). A tweezers‐like motion of the ATP‐binding cassette dimer in an ABC transport cycle. Molecular Cell, 12, 651–661. 14527411 10.1016/j.molcel.2003.08.004

[mbo3451-bib-0006] Chen, Q. , Yan, Q. P. , Wang, K. J. , Zhuang, Z. X. , & Wang, X. R. (2008). Portal of entry for pathogenic*Vibrio alginolyticus* into large yellow croaker *Pseudosciaena crocea*, and characteristics of bacterial adhesion to mucus. Diseases of Aquatic Organisms, 80, 181–188. 18814543 10.3354/dao01933

[mbo3451-bib-0007] Cortés, E. , Consuegra, J. , Sinisterra, R. , & Mendez‐Vilas, A. (2011). Biofilm formation, control and novel strategies for eradication. Science Against Microbial Pathogens: Communicating Current Research and Technological Advances, 2, 896–905.

[mbo3451-bib-0008] Daigle, F. , Graham, J. E. , & Curtiss, R. (2001). Identification of *Salmonella typhi* genes expressed within macrophages by selective capture of transcribed sequences (SCOTS). Molecular Microbiology, 41, 1211–1222. 11555299 10.1046/j.1365-2958.2001.02593.x

[mbo3451-bib-0009] Dunne, W. M. (2002). Bacterial adhesion: Seen any good biofilms lately? Clinical Microbiology Reviews, 15, 155–166. 11932228 10.1128/CMR.15.2.155-166.2002 PMC118072

[mbo3451-bib-0010] Espinosa‐Urgel, M. , Salido, A. , & Ramos, J. L. (2000). Genetic analysis of functions involved in adhesion of *Pseudomonas putida* to seeds. Journal of Bacteriology, 182, 2363–2369. 10762233 10.1128/jb.182.9.2363-2369.2000 PMC111295

[mbo3451-bib-0011] Foulongne, V. , Bourg, G. , Cazevieille, C. , Michaux‐Charachon, S. , & O'Callaghan, D. (2000). Identification of *Brucella suis* genes affecting intracellular survival in an in vitro human macrophage infection model by signature‐tagged transposon mutagenesis. Infection and Immunity, 68, 1297–1303. 10678941 10.1128/iai.68.3.1297-1303.2000 PMC97282

[mbo3451-bib-0012] Green, M. R. , & Sambrook, J. (2012). Molecular cloning: A laboratory manual. New York, NY: Cold Spring Harbor Laboratory Press.

[mbo3451-bib-0013] Grooteclaes, M. L. , & Frisch, S. M. (2000). Evidence for a function of CtBP in epithelial gene regulation and anoikis. Oncogene, 19, 3823–3828. 10949939 10.1038/sj.onc.1203721

[mbo3451-bib-0014] Grześkowiak, Ł. , Collado, M. C. , Vesterlund, S. , Mazurkiewicz, J. , & Salminen, S. (2011). Adhesion abilities of commensal fish bacteria by use of mucus model system: Quantitative analysis. Aquaculture, 318, 33–36.

[mbo3451-bib-0501] Herrero, M. , Delorenzo, V. , Timmis, K. N. (1990). Transposon vectors containing non‐antibiotic resistance selection markers for cloning and stable chromosomal insertion of foreign genes in gram‐negative bacteria. J. Bacteriol. 172, 6557‐6567. 2172216 10.1128/jb.172.11.6557-6567.1990 PMC526845

[mbo3451-bib-0015] He, Z. , Liang, J. , Tang, Z. , Ma, R. , Peng, H. , & Huang, Z. (2015). Role of the *luxs* gene in initial biofilm formation by *Streptococcus mutans* . Journal of Molecular Microbiology and Biotechnology, 25, 60–68. 25766758 10.1159/000371816

[mbo3451-bib-0016] Hori, K. , & Matsumoto, S. (2010). Bacterial adhesion: From mechanism to control. Biochemical Engineering Journal, 48, 424–434.

[mbo3451-bib-0017] Huang, L. , Hu, J. , Su, Y. , Qin, Y. , Kong, W. , Ma, Y. , … Yan, Q. (2015). Identification and characterization of three *Vibrio alginolyticus* non‐coding RNAs involved in adhesion, chemotaxis, and motility processes. Frontiers in Cellular and Infection Microbiology, 5, 1–13. 26217589 10.3389/fcimb.2015.00056 PMC4498440

[mbo3451-bib-0018] Huang, L. , Hu, J. , Su, Y. , Qin, Y. , Kong, W. , Zhao, L. , … Yan, Q. (2016). Genome‐wide detection of predicted non‐coding RNAs related to the adhesion process in *Vibrio alginolyticus* using high‐throughput sequencing. Frontiers in Microbiology, 7, 1–10. 27199948 10.3389/fmicb.2016.00619 PMC4848308

[mbo3451-bib-0019] Huang, L. , Qin, Y. , Yan, Q. , Lin, G. , Huang, L. , Huang, B. , & Huang, W. (2015). MinD plays an important role in *Aeromonas hydrophila* adherence to *Anguilla japonica* mucus. Gene, 565, 275–281. 25881868 10.1016/j.gene.2015.04.031

[mbo3451-bib-0020] Jaradat, Z. W. , & Bhunia, A. K. (2002). Glucose and nutrient concentrations affect the expression of a 104‐kilodalton Listeria adhesion protein in *Listeria monocytogenes* . Applied and Environmental Microbiology, 68(10), 4876–4883. 12324334 10.1128/AEM.68.10.4876-4883.2002 PMC126402

[mbo3451-bib-0021] Kim, Y. R. , & Rhee, J. H. (2003). Flagellar basal body flg operon as a virulence determinant of *Vibrio vulnificus* . Biochemical and Biophysical Research Communications, 304, 405–410. 12711330 10.1016/s0006-291x(03)00613-2

[mbo3451-bib-0022] Krovacek, K. , Pasquale, V. , Baloda, S. B. , Soprano, V. , Conte, M. , & Dumontet, S. (1994). Comparison of putative virulence factors in *Aeromonas hydrophila* strains isolated from the marine‐environment and human diarrheal cases in southern Italy. Applied and Environmental Microbiology, 60, 1379–1382. 8017924 10.1128/aem.60.4.1379-1382.1994 PMC201487

[mbo3451-bib-0023] Losensky, G. , Vidakovic, L. , Klingl, A. , Pfeifer, F. , & Fröls, S. (2015). Novel pili‐like surface structures of *Halobacterium salinarum* strain R1 are crucial for surface adhesion. Frontiers in Microbiology, 5, 755. 25628607 10.3389/fmicb.2014.00755 PMC4292770

[mbo3451-bib-0024] Luo, G. , Huang, L. , Su, Y. , Qin, Y. , Xu, X. , Zhao, L. , & Yan, Q. (2016). *flrA*,* flrB*, and *flrC* regulate adhesion by controlling the expression of critical virulence genes in *Vibrio alginolyticus* . Emerging Microbes & Infections, 5, e85. 27485498 10.1038/emi.2016.82 PMC5034100

[mbo3451-bib-0025] Martin, D. W. , & Mohr, C. D. (2000). Invasion and intracellular survival of *Burkholderia cepacia* . Infection and Immunity, 68, 24–29. 10603364 10.1128/iai.68.1.24-29.2000 PMC97097

[mbo3451-bib-0026] McCann, M. T. , Gilmore, B. F. , & Gorman, S. P. (2008). *Staphylococcus epidermidis* device‐related infections: Pathogenesis and clinical management. Journal of Pharmacy and Pharmacology, 60, 1551–1571. 19000360 10.1211/jpp/60.12.0001

[mbo3451-bib-0027] Mccoy, A. J. , Koizumi, Y. , Toma, C. , Higa, N. , Dixit, V. , Taniguchi, S. , et al. (2010). Cytotoxins of the human pathogen *Aeromonas hydrophila* trigger, via the NLRP3 inflammasome, caspase‐1 activation in macrophages. European Journal of Immunology, 40, 2797–2803. 20722078 10.1002/eji.201040490

[mbo3451-bib-0028] Nentwich, S. S. , Brinkrolf, K. , Gaigalat, L. , Huser, A. T. , Rey, D. A. , Mohrbach, T. , et al. (2009). Characterization of the laci‐type transcriptional repressor rbsr controlling ribose transport in corynebacterium glutamicum ATCC13032. Microbiology, 155, 150–164. 19118356 10.1099/mic.0.020388-0

[mbo3451-bib-0029] Ocallaghan, D. , Maskell, D. , Liew, F. Y. , Easmon, C. S. F. , & Dougan, G. (1988). Characterization of aromatic‐dependent and purine‐dependent salmonella‐typhimurium‐attenuation, persistence, and ability to induce protective immunity in BALB/c mice. Infection and Immunity, 56, 419–423. 3276625 10.1128/iai.56.2.419-423.1988 PMC259298

[mbo3451-bib-0030] Olga, S. , Jens, J. , & Michael, S. (2011). Virulence properties of the *Legionella pneumophila* cell envelope. Frontiers in Microbiology, 2, 74. 21747794 10.3389/fmicb.2011.00074 PMC3129009

[mbo3451-bib-0031] Qin, Y. X. , Lin, G. F. , Chen, W. B. , Huang, B. , Huang, W. S. , & Yan, Q. P. (2014). Flagellar motility contributes to the invasion and survival of *Aeromonas hydrophila* in *Anguilla japonica* macrophages. Fish & Shellfish Immunology, 39, 273–279. 24859591 10.1016/j.fsi.2014.05.016

[mbo3451-bib-0032] Rock, J. L. , & Nelson, D. R. (2006). Identification and characterization of a hemolysin gene cluster in *Vibrio anguillarum* . Infection and Immunity, 74, 2777–2786. 16622215 10.1128/IAI.74.5.2777-2786.2006 PMC1459744

[mbo3451-bib-0033] Shao, J. , Cheng, H. J. , Wang, C. Z. , Wu, D. Q. , Zhu, X. L. , Zhu, L. L. , Sun Z. , (2013). Sodium houttuyfonate, a potential phytoanticipin derivative of antibacterial agent, inhibits bacterial attachment and pyocyanine secretion of *Pseudomonas aeruginosa* by attenuating flagella‐mediated swimming motility. World Journal of Microbiology & Biotechnology, 29, 2373–2378. 23793941 10.1007/s11274-013-1405-2

[mbo3451-bib-0034] Shimada, T. , Kori, A. , & Ishihama, A. (2013). Involvement of the ribose operon repressor rbsr in regulation of purine nucleotide synthesis in *Escherichia coli* . FEMS Microbiology Letters, 344, 159–165. 23651393 10.1111/1574-6968.12172

[mbo3451-bib-0035] Sime‐Ngando, L. (2015). Aeromonas: A book review. Frontiers in Microbiology, 6, 106.

[mbo3451-bib-0036] Singer, T. , & Burke, E. (2003). High‐throughput TAIL‐PCR as a tool to identify DNA flanking insertions. Plant Functional Genomics, 236, 241–271. 10.1385/1-59259-413-1:241 14501069

[mbo3451-bib-0037] Skladchikova, G. , Ronn, L. C. , Berezin, V. , & Bock, E. (1999). Extracellular adenosine triphosphate affects neural cell adhesion molecule (NCAM)‐mediated cell adhesion and neurite outgrowth. Journal of Neuroscience Research, 57, 207–218. 10398298 10.1002/(SICI)1097-4547(19990715)57:2<207::AID-JNR6>3.0.CO;2-M

[mbo3451-bib-0038] Van Der Marel, M. , Schroers, V. , Neuhaus, H. , & Steinhagen, D. (2008). Chemotaxis towards, adhesion to, and growth in carp gut mucus of two *Aeromonas hydrophila* strains with different pathogenicity for common carp, *Cyprinus carpio* L. Journal of Fish Diseases, 31(5), 321–330. 18355183 10.1111/j.1365-2761.2008.00902.x

[mbo3451-bib-0039] Wang, L. , Huang, L. , Su, Y. , Qin, Y. , Kong, W. , Ma, Y. , … Yan, Q. (2015). Involvement of the flagellar assembly pathway in *Vibrio alginolyticus* adhesion under environmental stresses. Frontiers in Cellular and Infection Microbiology, 5, 1–9. 26322276 10.3389/fcimb.2015.00059 PMC4533019

[mbo3451-bib-0040] Yan, Q. P. , Chen, Q. , Ma, S. , Zhuang, Z. X. , & Wang, X. R. (2007). Characteristics of adherence of pathogenic *Vibrio alginolyticus* to theintestinal mucus of large yellow croaker (*Pseudosciaena crocea*). Aquaculture, 269, 21–30.

[mbo3451-bib-0041] Zhang, X. , Jiang, X. , Yang, L. , Fang, L. , Shen, H. , Lu, X. , Fang W. , (2015). DnaJ of *Streptococcus suis* type 2 contributes to cell adhesion and thermotolerance. Journal of Microbiology and Biotechnology, 25, 771–781. 25537722 10.4014/jmb.1408.08085

